# Establishment of reference values for plasma neurofilament light based on healthy individuals aged 5–90 years

**DOI:** 10.1093/braincomms/fcac174

**Published:** 2022-07-04

**Authors:** Joel Simrén, Ulf Andreasson, Johan Gobom, Marc Suarez Calvet, Barbara Borroni, Christopher Gillberg, Lars Nyberg, Roberta Ghidoni, Elisabeth Fernell, Mats Johnson, Herman Depypere, Caroline Hansson, Ingibjörg H Jonsdottir, Henrik Zetterberg, Kaj Blennow

**Affiliations:** Department of Psychiatry and Neurochemistry, Institute of Neuroscience and Physiology, Sahlgrenska Academy, University of Gothenburg, 41345 Gothenburg, Sweden; Clinical Neurochemistry Laboratory, Sahlgrenska University Hospital, 43180 Gothenburg, Sweden; Department of Psychiatry and Neurochemistry, Institute of Neuroscience and Physiology, Sahlgrenska Academy, University of Gothenburg, 41345 Gothenburg, Sweden; Clinical Neurochemistry Laboratory, Sahlgrenska University Hospital, 43180 Gothenburg, Sweden; Department of Psychiatry and Neurochemistry, Institute of Neuroscience and Physiology, Sahlgrenska Academy, University of Gothenburg, 41345 Gothenburg, Sweden; Clinical Neurochemistry Laboratory, Sahlgrenska University Hospital, 43180 Gothenburg, Sweden; Barcelonaβeta Brain Research Center (BBRC), Pasqual Maragall Foundation, 08005 Barcelona, Spain; IMIM (Hospital del Mar Medical Research Institute), 08003 Barcelona, Spain; Servei de Neurologia, Hospital del Mar, 08003 Barcelona, Spain; Centro de Investigación Biomédica en Red de Fragilidad y Envejecimiento Saludable (CIBERFES), 08003 Madrid, Spain; Centre for Neurodegenerative Disorders, Department of Clinical and Experimental Sciences, University of Brescia, 25125 Brescia, Italy; Gillberg Neuropsychiatry Centre, Sahlgrenska Academy, University of Gothenburg, 41119 Gothenburg, Sweden; Department of Integrative Medical Biology, Umeå University, 90736 Umeå, Sweden; Umeå Center for Functional Brain Imaging (UFBI), Umeå University, 90736 Umeå, Sweden; Department of Radiation Sciences, Umeå University, 90185 Umeå, Sweden; Molecular Markers Laboratory, IRCCS Istituto Centro San Giovanni di Dio Fatebenefratelli, 25125 Brescia, Italy; Gillberg Neuropsychiatry Centre, Sahlgrenska Academy, University of Gothenburg, 41119 Gothenburg, Sweden; Gillberg Neuropsychiatry Centre, Sahlgrenska Academy, University of Gothenburg, 41119 Gothenburg, Sweden; Department of Gynecology, Ghent University Hospital, B-9820 Ghent, Belgium; Department of Psychiatry and Neurochemistry, Institute of Neuroscience and Physiology, Sahlgrenska Academy, University of Gothenburg, 41345 Gothenburg, Sweden; The Institute of Stress Medicine, Region of Västra Götaland, 41319 Gothenburg, Sweden; The Institute of Stress Medicine, Region of Västra Götaland, 41319 Gothenburg, Sweden; School of Public Health and Community Medicine, Institute of Medicine, Sahlgrenska Academy, University of Gothenburg, 41319 Gothenburg, Sweden; Department of Psychiatry and Neurochemistry, Institute of Neuroscience and Physiology, Sahlgrenska Academy, University of Gothenburg, 41345 Gothenburg, Sweden; Clinical Neurochemistry Laboratory, Sahlgrenska University Hospital, 43180 Gothenburg, Sweden; Department of Neurodegenerative Disease, Institute of Neurology, University College London, WC1E 6BT London, UK; UK Dementia Research Institute, University College London, WC1E 6BT London, UK; Hong Kong Center for Neurodegenerative Diseases, Hong Kong, China; Department of Psychiatry and Neurochemistry, Institute of Neuroscience and Physiology, Sahlgrenska Academy, University of Gothenburg, 41345 Gothenburg, Sweden; Clinical Neurochemistry Laboratory, Sahlgrenska University Hospital, 43180 Gothenburg, Sweden

**Keywords:** neurofilament light, plasma, biomarkers, reference limits, clinical chemistry

## Abstract

The recent development of assays that accurately quantify neurofilament light, a neuronal cytoskeleton protein, in plasma has generated a vast literature supporting that it is a sensitive, dynamic, and robust biomarker of neuroaxonal damage. As a result, efforts are now made to introduce plasma neurofilament light into clinical routine practice, making it an easily accessible complement to its cerebrospinal fluid counterpart. An increasing literature supports the use of plasma neurofilament light in differentiating neurodegenerative diseases from their non-neurodegenerative mimics and suggests it is a valuable biomarker for the evaluation of the effect of putative disease-modifying treatments (e.g. in multiple sclerosis). More contexts of use will likely emerge over the coming years. However, to assist clinical interpretation of laboratory test values, it is crucial to establish normal reference intervals. In this study, we sought to derive reliable cut-offs by pooling quantified plasma neurofilament light in neurologically healthy participants (5–90 years) from eight cohorts. A strong relationship between age and plasma neurofilament light prompted us to define the following age-partitioned reference limits (upper 95^th^ percentile in each age category): 5–17 years = 7 pg/mL; 18–50 years = 10 pg/mL; 51–60 years = 15 pg/mL; 61–70 years = 20 pg/mL; 70 + years = 35 pg/mL. The established reference limits across the lifespan will aid the introduction of plasma neurofilament light into clinical routine, and thereby contribute to diagnostics and disease-monitoring in neurological practice.

## Teaser

Simrén *et al.* report age-stratified cut-offs for plasma neurofilament light, based on a large material of healthy individuals across the ages 5–90 years. The findings will assist clinical implementation of plasma neurofilament light in clinical routine, by simplifying interpretation of concentrations across the lifespan as neurofilament light increases with age.

## Graphical Abstract

**Graphical abstract:**
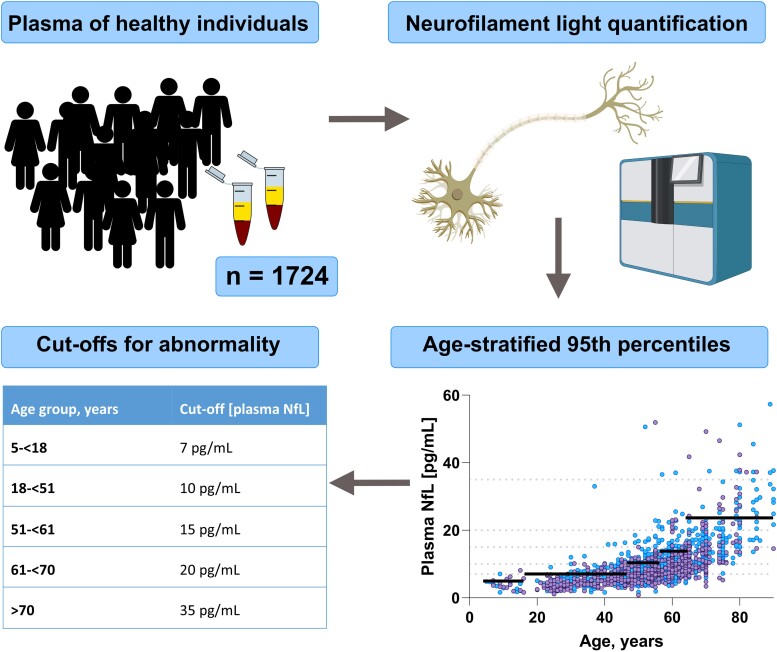

## Introduction

In recent years, there has been rapid progress in the field of fluid biomarkers for brain disorders, facilitated by the advent of ultrasensitive techniques with capabilities of quantifying proteins at sub-femtomolar concentrations.^1^ This has enabled many of the biomarkers that were previously only possible to measure in CSF to be translated to blood. One of the best examples of this transition is neurofilament light chain (NfL), a neuroaxonal intermediate filament protein, which is highly expressed especially in large myelinated axons,^2^ and is important for axonal stability and growth.^3^ In response to neuroaxonal damage, it is released into the CSF as well as the bloodstream.^3^ The successful development of a blood assay was first demonstrated in HIV infected patients,^4^ adopting the Simoa technique.^1^ The strong correlation with CSF NfL (R∼0.8–0.9)^4,5^ and the capability to quantify the very low concentrations found in normal individuals sparked interest in the assay, which then led to a vast literature reporting increased levels of plasma NfL in multiple diseases of the nervous system.^3^ These include both acute neurological conditions, such as traumatic and vascular brain injury,^6,7^ infections^4^ as well as in neurodegenerative and neuroinflammatory conditions.^8,9^ Notable examples include multiple sclerosis, for which NfL is now starting to be used as a biomarker of disease activity and therapeutic response.^10^ Furthermore, it can be used to strengthen the differential diagnosis between idiopathic Parkinson's disease and atypical Parkinsonian disorders, as well as between neurodegenerative diseases and non-neurodegenerative mimics.^9^ In addition, the concentrations remain stable over shorter time periods in normal individuals,^11^ and are resistant to alterations in preanalytical sample handling, such as repeated freeze-thaw cycles^12,13^ and delayed processing.^14^

The evidence suggesting that NfL is a dynamic, pre-analytically and analytically robust biomarker of neuroaxonal damage has led to an increasing interest in introducing plasma NfL measurements into clinical laboratory routine. One important step towards realizing this goal is to establish cut-offs, which is commonly attained by determining the concentrations in a healthy reference sample. Since studies report a marked age-dependent increase of plasma NfL,^15^ which is also well known for NfL in CSF,^16^ we sought to measure NfL in healthy individuals across the life span to derive reliable cut-offs to be used in clinical neurochemistry routine.

## Methods

### Study design and population

For this study, we collected data from eight cohorts, spanning the ages of 5–90 where the subjects had no history or clinical symptoms or signs of neurologic disorder. The selection was made to maximize the clinical relevance of the reference ranges, by including a sufficient number of individuals from research in which we had NfL quantified in blood across the largest possible age-span. The first cohort included children from the Child Neuropsychiatry Centre (CNC)/Gillberg Neuropsychiatry Centre in Gothenburg, Sweden (cohort one, ‘CNC’, age range 5–16 years), among which 10 were controls in the study and 17 had a neuropsychiatric illness, but with NfL concentrations which did not differ from the control group.^17^ The second cohort consisted of individuals from the ALFA+ study, which was established at the Barcelona βeta Brain Research Center, Barcelona, Spain to study the preclinical features of Alzheimer's disease. From this study, we only included participants with no evidence of Aβ pathology, as measured by CSF Aβ42/40, with a cut-off previously established^18^ (cohort two, ‘ALFA+’, age range: 50–68 years).^19^ Furthermore, we included individuals from a study at the Institute for Stress Medicine in which consisted of individuals with symptoms of exhaustion disorder, but without any neurological comorbidities (*n* = 150) which had measurements at baseline and at a second visit (range 7–12 years apart), and healthy controls (*n* = 100) (cohort three, ‘Stress’, age range: 21–74 years). The fourth cohort consisted of individuals from the Swedish longitudinal Betula study, which is a population-based prospective study on aging, memory, and dementia conducted in Umeå, Sweden. Participants with dementia were excluded (cohort four, ‘Betula’, age range: 44–90 years).^20^ The fifth cohort encompassed the baseline blood samples from healthy individuals from an intervention study at the Department of Gynaecology, Ghent University Hospital, Ghent, Belgium, investigating the effects of menopause in women (cohort five, ‘Estrogen’, age range: 34–67 years). In addition, healthy individuals between 20–75 years of age provided plasma samples at the blood donation facility at Sahlgrenska University hospital, Gothenburg, Sweden. These individuals reported being at good general health, and passed the extensive screening required to donate blood in Sweden (cohort six, ‘Blood donors’, age range: 20–75 years). Furthermore, we included healthy young men from a study where the effects of sleep deprivation on biomarkers were investigated at Sahlgrenska University Hospital, Mölndal, Sweden (cohort seven, ‘Sleep’, age range: 21–35 years).^21^ Finally, we included healthy controls from a biomarker study at the University of Brescia, Brescia, Italy. These participants were recruited among spouses or caregivers and underwent a brief standardized neuropsychological assessment [Mini Mental State Examination (MMSE) ≥27/30]; psychiatric or other neurological illnesses were considered exclusion criteria (cohort eight, ‘Brescia’, age range: 29–83 years).^22^ The study was conducted according to the Declaration of Helsinki and approved by the local ethics committees at each participating site.

### Biochemical analysis

Blood was collected by venipuncture in ethylenediaminetetraacetic acid (EDTA) tubes and was centrifuged within two hours of collection at 20°C, centrifuged at 2000 g for 10 min and stored at –80°C pending biochemical analysis in cohorts 2–7. The exact time-interval between these procedures are not known to us. However, we have previously shown that delayed centrifugation has very limited impact on the stability of NfL measured in EDTA-plasma.^14^ All samples were measured in plasma, except for in cohort one and eight, in which serum was used. In those cohorts, blood was collected in serum-separating tubes, which was left to coagulate for 30 min before the sample was centrifuged and stored as previously mentioned pending analysis. NfL was measured in all samples using commercial Quanterix® kits (Simoa® NF-light Kit) on Simoa HD-X or HD-1 analyzers according to the manufacturer's instructions (Quanterix, Billerica, MA, USA). Samples were run in singlicate and each assay plate included internal quality control (QC) samples with high and low plasma NfL concentrations, respectively, analyzed in duplicate both in the beginning and end of the plate. Since evidence suggest that NfL in serum is consistently ∼20% higher than EDTA plasma,^12^ all serum concentrations were divided by 1.20 to harmonize with plasma concentrations. Two clear outliers with concentrations of 272 pg/mL and 397 pg/mL, respectively, were excluded from the final sample.

### Normalization procedure in clinical chemistry routine

Another plasma pool, run in the same way as the QCs, has been introduced to the protocol used in the clinical routine analysis at the Clinical Neurochemistry Laboratory, Sahlgrenska University Hospital, Mölndal, Sweden. This sample, named internal calibrator (IC), has an assigned concentration, determined during the validation of the assay, and is measured in quadruplicate in each analytical run and used for normalization. Each run is approved based on the Westgard rules^23^ 1_3s_ (no QCs deviate more than 3 standard deviations (SD) from the QC mean) and 2_2s_ (not more than two QCs are allowed to deviate more than 2 SD from the QC mean) for the normalized QCs. Conceptually, the use of an IC as described is similar to how random-access instruments are programmed with a master calibration curve, built on many calibrators with different concentrations, which is then adjusted based on only a couple of calibrators included in each analytical run. This is performed to achieve longitudinal stability of the assay across batches and other potential factors which may be influencing the performance over time.

### Statistical analysis

Determination of distribution was conducted by inspecting histograms of the data. Since there was a visible positive skewness of NfL concentrations, the data was log_10_ transformed for statistical analyses. For group comparisons, student's *t*-test was used. Linear regression models were used to assess the relationship of NfL with age, and to estimate the proportion of the variance in NfL explained by age and sex, respectively. A local weighted regression (LOESS) plot was generated to visualize the changing NfL concentrations across the lifespan.^24^ As it is not believed that low concentrations of NfL are associated with pathology, the upper 95th percentile was estimated using a rank-based method, as recommended by the International federation for Clinical Chemistry and Laboratory Medicine (IFCC).^25^ We performed a sensitivity analysis, excluding the participants from cohort three, and comparing if this significantly affected the mean NfL concentrations in the relevant age-ranges (19–50, 51–60, 61–70 and >70). Since no significant mean differences were seen when excluding the participants from cohort three, they were included in the determination of the reference values. A summary table of the results can be seen in the Supplementary material (Supplementary Table 1). There were no missing data in this study. Statistical analyses and graphs were generated using GraphPad Prism v9.0 (La Jolla, CA) or SPSS (v. 27.0). Test results with a two-sided *P* < 0.05 were considered significant.

### Data availability

All data are available upon reasonable request.

## Results

### Demographic characteristics

**Figure 1:**
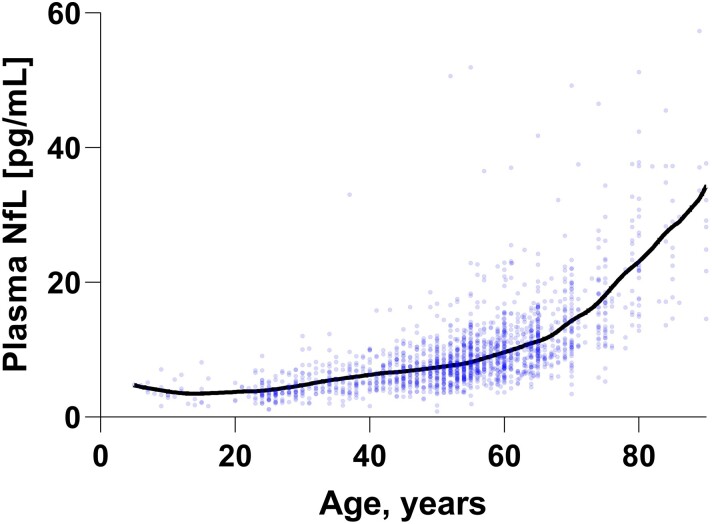
**The age-related increase of plasma NfL accelerates across the lifespan.** The increasing NfL concentrations with age are visualized using a LOESS plot (locally estimated scatterplot smoothing) between ages 5–90. Three data points with concentrations 63.9, 82.6, and 77.7 pg/mL are not shown for visualization purposes, but were included in all statistical analyses in the paper.

In total, 1724 measurements were included, among which 1104 (64%) were from women. For description of each cohort, see Table 1. Plasma NfL concentrations increased significantly with increasing age (*r*^2^ = 0.53, β = 0.012, *P* < 0.001). This increase is seemingly more prominent after ∼65 years of age (Fig. 1). The age [mean years (SD)] of the women across the cohorts included were slightly higher than the male participants [54.9 (13.5) versus 51.2 (16.0), 95% confidence interval (CI) (2.3–5.1)] and had slightly higher concentrations of plasma NfL (10.1 versus 8.9 pg/mL, 95% CI [0.47–1.8], both *P* < 0.001). However, when including both factors in a regression model, age was much more strongly associated with NfL concentrations than sex (age: β = 0.73, *P* = 0.001; sex: β = −0.041, *P* = 0.014).

**Table 1:** Cohort characteristics

Cohort	Name	Measurements, *n*	Mean age, years (SD)	Sex, females/male (% females)
Cohort 1	CNC	27	11.0 (3.3)	12/15 (44%)
Cohort 2	ALFA+	184	60.2 (4.5)	124/60 (67%)
Cohort 3	Stress	397	45.7 (11.5)	284/113 (72%)
Cohort 4	Betula	407	66.0 (10.7)	207/200 (51%)
Cohort 5	Estrogen	321	54.2 (4.7)	321/0 (100%)
Cohort 6	Blood donors	304	45.5 (13.4)	104/198 (35%)
Cohort 7	Sleep	21	25.4 (3.3)	0/21 (0%)
Cohort 8	Brescia	63	65.4 (12.1)	50/13 (79%)
Total		1724	53.5 (14.5)	1104/620 (64%)

### Derivation of age-stratified cut-offs

**Figure 2:**
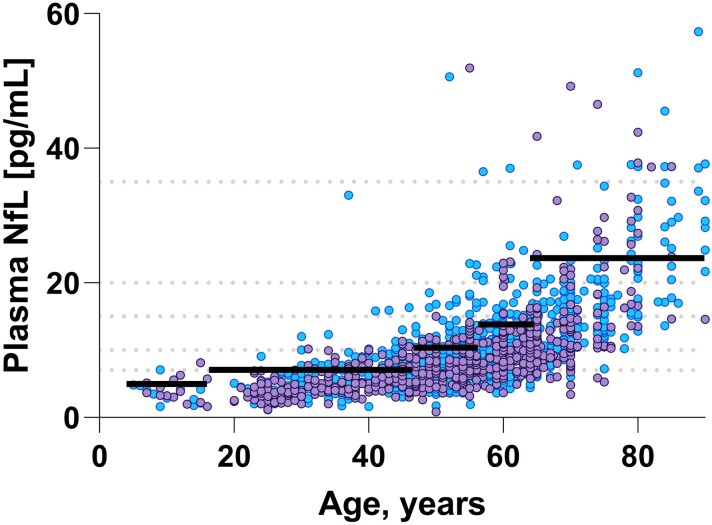
**Age-stratified cut-offs for plasma neurofilament light.** This graph displays the age-dependent increase in plasma NfL concentration in individuals aged 5–90. The black solid lines, as well as the grey dashed lines represent the cut-offs derived from a rank-based method estimating the 95th percentile in each age category. The estimated 95^th^ percentiles are shown in brackets after the respective age-categories: (i) 5–17 (7 pg/mL), (ii) 18–50 (10 pg/mL), (iii) 51–60 (15 pg/mL), (iv) 61–70 (20 pg/mL), and (v) >70 (35 pg/mL). Male individuals are indicated by purple dots, and females with blue dots. Three data points with concentrations 63.9, 82.6, and 77.7 pg/mL are not shown for visualization purposes, but were included in all statistical analyses in the paper.

Since we and others previously have reported age-dependent increases in NfL, we determined age-specific cut offs for the following age categories by visually inspecting a scatter plot where plasma NfL was regressed against age: 5 to <18 (*n* = 27), 18 to <51 (*n* = 369), 51 to <60 (*n* = 614), 61 to <71 (*n* = 366), >70 (*n* = 150). Based on the 95th percentile in each of the groups, the following reference limits were established: 7 pg/mL in the group between ages 5–17, 10 pg/mL between the ages of 18–50, 15 pg/mL in the ages ranging from 51–60, 20 pg/mL in the group ranging between 61–70, and 35 pg/mL for individuals older than 70 years (Fig. 2 and Table 2).

**Table 2:** Age-specific cut-offs for plasma NfL

Age group, years	Measurements, n.	Plasma NfL (pg/mL), mean (SD)	Cut-off (plasma NfL)
5 to <18	27	3.89 (1.67)	7 pg/mL
18 to <51	568	6.07 (2.85)	10 pg/mL
51 to <61	614	8.92 (4.21)	15 pg/mL
61 to <70	365	12.0 (5.20)	20 pg/mL
>70	150	21.9 (12.1)	35 pg/mL

### Achieving traceability to the reference ranges

As an example of how the normalization affects the dispersion of the QCs included in each analytical run, 20 randomly picked runs from a time period of >3 months, resulted in a decrease in the intermediate precision from 7.2% to 6.1% and 13% to 11% for the high and low QCs, respectively. Laboratories that plan to introduce plasma NfL in clinical routine practice are welcome to send samples to the Clinical Neurochemistry Laboratory at Sahlgrenska University Hospital, Mölndal, Sweden, to achieve traceability to the data presented here.

## Discussion

In this study, we present a large material of normal plasma NfL concentrations from a large number of healthy participants spanning the ages 5–90 years. We derived specific cut-offs in five different age categories to reflect the clear effect of age on concentrations of plasma NfL. A clear effect of age was also reported in a study where Hviid *et al.*^13^ aimed to establish reference ranges for NfL. However, in that study, which used serum as the reference matrix, the proposed cut-offs was significantly higher, especially in the group of individuals above the age of 65 (n = 60) (<53 pg/mL versus <35 pg/mL for individuals >70 in our study)^13^, possibly reflecting a larger degree of sub- or preclinical neuroaxonal pathology in their study, the use of a different matrix^12^ and also the smaller sample size. Furthermore, since the study by Hviid *et al.*^13^ only included individuals >18, we are the first to report that NfL in children is lower than in young adults (7 pg/mL <18 years versus 10 pg/mL 18–41 years). The establishment of cut-offs in children also provides an opportunity to identify neuroaxonal injury to assist clinical diagnosis and monitor neurological disease also in pediatric patients. An example where this has been successful is spinal muscular atrophy, where CSF NfL normalizes in response to treatment.^26^

NfL has the potential to be widely used both in primary care as well as in secondary and tertiary settings as a diagnostic marker of apparent damage to the central nervous system, as well as to monitor treatment response and disease progression. Examples of the diagnostic utility of NfL is its ability to differentiate between idiopathic Parkinson's disease and atypical parkinsonian syndromes,^5,9^ as well as between primary psychiatric syndromes and frontotemporal dementia, where levels are roughly five-fold higher.^9,27^ However, as with most clinical chemistry analyses, changes due to the normal biology exist also for NfL. In normal individuals, there is a significant inter-individual variability, which needs to be considered when interpreting plasma NfL.^11^ Nonetheless, the normal intra-individual variability over a short time frame (days) is almost negligible, making repeated measures of NfL reliable.^11^ A recent study suggested that an intra-individual change of 24.3% can be considered significant on an individual level, based on biological and analytical variation.^11^ Thus, it is likely that the increases that are seen over time (likely over one/a few years rather than weeks or months) in neurodegenerative conditions with a relatively fast progression can be longitudinally monitored in a reliable manner, aiding clinical management.^28–30^ As mentioned above, NfL has the potential to be used in the monitoring of pharmacological therapy, which has been robustly demonstrated in patients with multiple sclerosis where levels normalize in response to treatment with disease modifying therapies.^31^ As novel pharmacological treatments that intervene with the natural course of neurological diseases, the accessibility and relatively low cost of NfL entails a large clinical utility.

Strengths of this study include the large number of participants across the lifespan, providing an opportunity to derived reliable age-partitioned reference limits for clinical use. Furthermore, all individuals in this study had concentrations above the lower limit of quantification, reflecting the large dynamic range of the assay, which allows for monitoring also of participants that are ‘low producers’. Another strength of this study is that all participants had NfL quantified using Simoa, which is by far the most used method to quantify NfL in blood. Efforts are currently ongoing within the IFCC to harmonize measurements across several analytical platforms which, after adjustment to reference materials, will make it possible to generalize these reference ranges also to other platforms.

Limitations of this study include the relatively small number of children included in this study – a higher number of participants below the age of 18 would increase the certainty of the reference limit for these groups of individuals. Still, the NfL concentration in these age-groups were very narrowly distributed, and we believe that this reflects the absence of clinically silent comorbidities that give rise to higher NfL concentrations in plasma. In addition, we did not have any values for children between zero and five years of age, but our clinical experience is that the levels in this age-span is not significantly different from older children, unlike tau, which is present in very high levels in CSF during early brain development and the first year of life.^32^ Furthermore, in most of the cohorts included, we did not have the possibility to perform advanced imaging and thus exclude participants with sub- or preclinical neuronal injury. This may be reflected in the greater variability of plasma NfL concentrations with increasing age, as seen in Fig. 1. Another possible confounder is the effect of low body mass index, which has been reported to be associated with increased plasma NfL concentration in some recent publications.^10,33^ According to Benkert *et al.*,^10^ however, this effect was small, explaining around 5% of the total variance of plasma NfL. Thus, we believe that clinically meaningful changes in neuroaxonal integrity are still found using these reference limits. To conclude, here we present the largest material of normal values of NfL across the lifespan to derive age-partitioned reference values, greatly aiding the clinical implementation of NfL.

## Supplementary Material

fcac174_Supplementary_Data

## Abbreviations

CNC =: child neuropsychiatry center
EDTA =: ethylenediaminetetraacetic acid
IFCC =: International Federation for Clinical Chemistry and Laboratory Medicine
MMSE =: mini mental state examination
NfL =: neurofilament light
QC =: quality control
