# Correction to: Establishment of reference values for plasma neurofilament light based on healthy individuals aged 5–90 years

**DOI:** 10.1093/braincomms/fcag159

**Published:** 2026-05-08

This is a correction to: Joel Simrén, Ulf Andreasson, Johan Gobom, Marc Suarez Calvet, Barbara Borroni, Christopher Gillberg, Lars Nyberg, Roberta Ghidoni, Elisabeth Fernell, Mats Johnson, Herman Depypere, Caroline Hansson, Ingibjörg H Jonsdottir, Henrik Zetterberg, Kaj Blennow, Establishment of reference values for plasma neurofilament light based on healthy individuals aged 5–90 years, *Brain Communications*, Volume 4, Issue 4, 2022, fcac174, https://doi.org/10.1093/braincomms/fcac174

In the originally published version of this manuscript, the solid black lines in Figure 2 which represent cut-offs derived from a rank-based method estimating the 95th percentile in each age category were incorrectly placed. This affected both Figure 2 and the Graphical Abstract for the article. The corrected figures are included here.

Figure 2:

**Figure:**
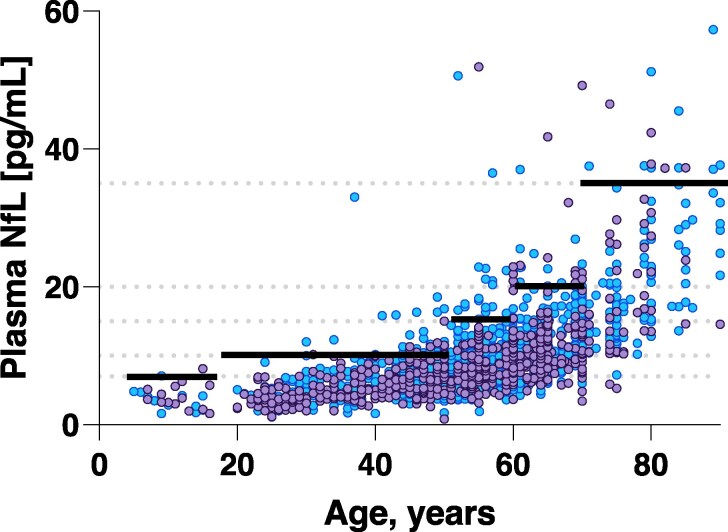


Graphical Abstract:

**Figure:**
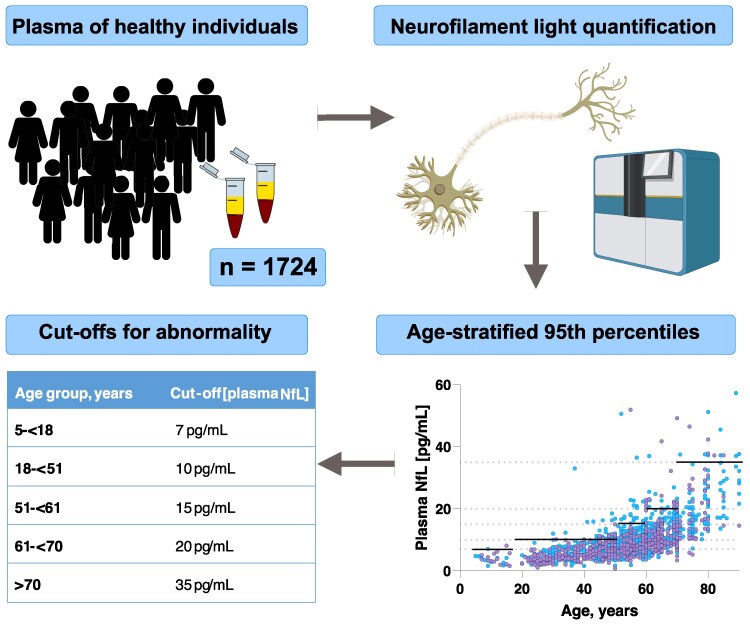


These details have been corrected only in this correction notice to preserve the published version of record.

