# Supplementary material for: Establishment of reference values for plasma neurofilament light based on healthy individuals aged 5–90 years
Source: Brain Commun. 2022 Jul 4;4(4):fcac174. doi: 10.1093/braincomms/fcac174 (PMC9297091; doi:10.1093/braincomms/fcac174)
Supplement: fcac174_Supplementary_Data [file fcac174_supplementary_data.docx]

**Supplementary Material**

*Simrén et al.* Establishment of reference values for plasma neurofilament light based on healthy individuals aged 5-90 years.

**Supplementary table 1.** Sensitivity analysis of excluding cohort three.

| Age group | All individuals | | Excluding cohort three | | *P*-value |
| --- | --- | --- | --- | --- | --- |
|  | n. | NfL (pg/mL), mean (SD) | n. | NfL (pg/mL), mean (SD) |  |
| 19–50 | 568 | 6.07 (2.84) | 305 | 5.73 (2.56) | 0.08 |
| 51–60 | 614 | 8.92 (4.21) | 525 | 8.81 (4.29) | 0.49 |
| 61–70 | 365 | 12.01 (5.20) | 326 | 11.94 (5.23) | 0.86 |
| >70 | 150 | 21.92 (12.06) | 144 | 22.2 (12.17) | 0.87 |

The table shows the comparisons of individuals across age groups. All *P*-values were derived from T-tests of log_10_ transformed data. Abbreviations; NfL, neurofilament light.
